# Exploring Medical Student’s Attitudes to Electroconvulsive Therapy (ECT)

**DOI:** 10.1192/bjo.2026.11306

**Published:** 2026-06-30

**Authors:** Isabel Schlegel, Georgia Wylie, Julie Langan Martin

**Affiliations:** 1 University of Glasgow, Glasgow, United Kingdom; 2 NHS Ayrshire and Arram, Glasgow, United Kingdom

## Abstract

**Aims:**

Electroconvulsive therapy (ECT) is an effective and safe treatment for severe mental illnesses yet it remains stigmatised with widespread misconceptions- even among healthcare professionals and medical students. Previous studies have reported that medical students often have limited and inaccurate knowledge about ECT, particularly regarding itsadministration and safety. The aim of our study was to explore medical students’ attitudes to ECT before they engaged with a Technology-Enhanced Learning and Teaching (TELT) resource which focused on ECT.

**Methods:**

An online survey was sent to all medical students during their clinical placement in psychiatry to explore their knowledge and attitudes about ECT. The questionnaire was adapted from those used in other studies to explore knowledge and attitudes towards ECT. Ethical approval was obtained from the MVLS Ethics Committee as was approval from the Head of the Undergraduate Medical School.

**Results:**

To date, 104 students have completed the pre-teaching questionnaire. Of the respondents,57% (n=59) identified as female and 40% (n=41) identified as male.The majority of respondents (70%, n=72) reported that a friend or relative had been diagnosed with a mental health problem and 16% (n=16) reported that they themselves, had been diagnosed with a mental illness.

Just under half (47%, n=48) stated that they would consider specialising in psychiatry. Respondents reported that prior knowledge about ECT came from movies and TV (67%, n=70), the internet (53%, n=55) and medical books and journals (37%, n=38). Baseline knowledge of ECT was limited: only 64% (n=66) were aware a general anaesthetic was given and only half (51%, n=53) were aware that muscle relaxants were used. Only 33% (n=34) recognised that ECT did not cause permanent brain damage and 38% (n=40) were aware that ECT did not cause burns. Moreover, 65% (n=68) reported they would be afraid to receive ECT themselves and 59% (n=61) reported that their view of ECT had been negatively impacted by the media.

**Conclusion:**

Despite respondents reporting high rates of exposure to mental health problems in family and friends and high rates of respondents considering a career in psychiatry, baseline knowledge of ECT was very limited, and appeared to be influence by the media. Misconceptions around the procedure, were relatively common with many respondents expressing fear over receiving the treatment themselves. These results highlight the need for educational resources to address misconceptions, improve knowledge and counteract the negative influence of media depictions of ECT.

